# Sex-stratified Genomic Structural Equation Models of Posttraumatic Stress Inform PTSD Etiology: L'utilisation de la modélisation génomique par équations structurelles stratifiée par sexe du stress post-traumatique pour expliquer l'étiologie du TSPT

**DOI:** 10.1177/07067437241301016

**Published:** 2024-12-09

**Authors:** Ashley Moo-Choy, Murray B. Stein, Joel Gelernter, Frank R. Wendt

**Affiliations:** 1 Department of Anthropology, 7938 University of Toronto, Toronto, ON, Canada; 2 Forensic Science Program, 7938 University of Toronto, Mississauga, ON, Canada; 3 Krembil Centre for Neuroinformatics, 7978 Centre for Addiction and Mental Health, Toronto, ON, Canada; 4 VA San Diego Healthcare System, Psychiatry Service, San Diego, CA, USA; 5 Department of Psychiatry, 8784 University of California San Diego, La Jolla, CA, USA; 6 Herbert Wertheim School of Public Health and Human Longevity Science, 8784 University of California San Diego, La Jolla, CA, USA; 7 Department of Psychiatry, 12228 Yale School of Medicine, New Haven, CT, USA; 8 VA CT Healthcare System, West Haven, CT, USA; 9 Department of Genetics, 12228 Yale School of Medicine, New Haven, CT, USA; 10 Department of Neuroscience, 12228 Yale School of Medicine, New Haven, CT, USA; 11 Biostatistics Division, Dalla Lana School of Public Health, 7938 University of Toronto, Toronto, ON, Canada

**Keywords:** PTSD, posttraumatic stress, genomic structural equation models, sex differences

## Abstract

**Objective:**

Posttraumatic stress disorder (PTSD) affects 3.9%–5.6% of the worldwide population, with well-documented sex-related differences. While psychosocial and hormonal factors affecting sex differences in PTSD and posttraumatic stress (PTS) symptom etiology have been explored, there has been limited focus on the genetic bases of these differences. Many symptom combinations may confer a PTSD diagnosis. We hypothesized that these symptom combinations have sex-specific patterns, the examination of which could inform etiological differences in PTSD genetics between males and females.

**Methods:**

To investigate this, we performed a sex-stratified multivariate genome-wide association study (GWAS) in unrelated UK Biobank (UKB) individuals of European ancestry. Using GWAS summary association data, genomic structural equation modelling was performed to generate sex-specific factor models using 6 indicator variables: trouble concentrating, feeling distant from others, irritability, disturbing thoughts, upset feelings, and avoidance of places/activities which remind the individual of a traumatic event.

**Results:**

Models of male and female PTSD symptoms differed substantially (local standardized root mean square difference = 3.12) and significantly (χ^2^(5) = 28.03, *P* = 3.6 × 10^−5^). Independent 2-factor models best fit the data in both males and females; these factors were subjected to GWAS in each sex, revealing 3 genome-wide significant loci in females, mapping to *SCAND3*, *WDPCP*, and *FAM120A*. No genome-wide significant loci were identified in males. All 4 PTS factors (2 in males and 2 in females) were heritable.

**Conclusions:**

By assessing the relationship between sex and PTSD symptoms, this study informs correlative and putatively causal etiological differences between males and females which support further investigation of sex differences in PTSD genetics.

## Résumé

**Objectif:**

le trouble de stress post-traumatique (TSPT) affecte de 3,9 % à 5,6 % de la population mondiale, les différences liées au sexe étant bien documentées. Bien que les facteurs psychosociaux et hormonaux affectant les différences entre hommes et femmes dans l'étiologie des symptômes du TSPT et du stress post-traumatique (SPT) aient été étudiés, les bases génétiques de ces différences n'ont pas fait l'objet d'une attention particulière. De nombreuses associations de symptômes peuvent conduire à un diagnostic de TSPT. Nous avons émis l'hypothèse que ces associations de symptômes présentent des caractéristiques propres à chaque sexe, dont l'examen pourrait nous éclairer sur les différences étiologiques dans la dimension génétique du TSPT entre les hommes et les femmes.

**Méthodologie:**

pour étudier cette question, nous avons réalisé une étude d'association pangénomique (GWAS) multivariée stratifiée selon le sexe chez des individus non apparentés d'ascendance européenne figurant dans la base de données UK Biobank (UKB). À partir des données d'association sommaires issues de la GWAS, une modélisation génomique par équations structurelles a été réalisée pour générer des modèles factoriels par sexe à l'aide de six variables indicatrices : difficultés de concentration, sentiment de détachement par rapport aux autres, irritabilité, pensées perturbantes, sentiments de contrariété et évitement des lieux/activités qui rappellent à l'individu un événement traumatisant.

**Résultats:**

les modèles de symptômes de TSPT chez les hommes et les femmes différaient considérablement (différence quadratique moyenne standardisée locale = 3,12) et de manière significative (^2^(5)=28,03, p = 3,6 x 10^-5^). Des modèles indépendants à deux facteurs conviennent le mieux aux données chez les hommes et les femmes; ces facteurs ont été soumis à une GWAS dans chaque sexe, révélant trois locus pangénomiques significatifs chez les femmes, correspondant à *SCAND3*, *WDPCP* et *FAM120A*. Aucun locus pangénomique significatif n'a été identifié chez les hommes. Les quatre facteurs de SPT (deux chez les hommes et deux chez les femmes) étaient héréditaires.

**Conclusions:**

en évaluant la relation entre le sexe et les symptômes de TSPT, cette étude fournit des informations sur les différences étiologiques corrélatives et potentiellement causales entre les hommes et les femmes, ce qui justifie des recherches plus approfondies sur les différences entre les sexes reliées à la dimension génétique du TSPT.

## Introduction

Posttraumatic stress disorder (PTSD) affects an estimated 3.9%–5.6% of the worldwide population.^1^ In individuals with severe mental illness, an estimated 30% also suffer from PTSD.^2^ The Diagnostic and Statistical Manual of Mental Disorders, 5th Edition (DSM-5) defines traumatic exposure as experiencing, witnessing, and/or learning of a traumatic event involving friends or family, or by repetitive personal exposure.^3^ Diagnosis of PTSD after trauma exposure requires an individual to experience intrusive memories of the traumatic event(s), hyperarousal, avoiding reminders of trauma, and negative alterations in cognition and mood related to the trauma for a period of at least 1 month, resulting in significant distress and/or functional impairment.

Trauma exposure and PTSD are heritable, meaning genetic factors explain a portion of trait variation,^4^ while affecting people differently based on sex, gender identity, and age at exposure.^5,6^ Women have at least 2 times higher lifetime risk of PTSD, in part due to more common exposure to different types of trauma than men (e.g., sexual assault and intimate partner violence) and age at trauma exposure (e.g., childhood sexual abuse). There have been both biological and psychosocial hypotheses as to the cause of sex-based differences in PTSD onset, symptomatology, and treatment, but sex-based empirical biological data are lacking.^4^ Some studies suggest that the increased prevalence of PTSD in women is due to different PTSD symptomatology relative to men.^7^ For example, women report more re-experiencing symptoms than men, motivating the exploration of different etiologies and patterns of symptom clustering.

Based on genome-wide association studies (GWAS), the SNP-based heritability of PTSD in women has been consistently non-zero and detectable even in early studies of PTSD with limited sample size.^8,9^ Conversely, studies in men required larger sample sizes to detect a significant SNP-based heritability estimate.^8,9^ Independent of sex and/or gender differences in PTSD presentation, PTSD genetics correlates with genetic factors influencing depression,^10^ anxiety,^11^ attention deficit hyperactivity disorder,^12^ schizophrenia,^8^ and bipolar disorder,^13^ among others. Prior reports postulate that various combinations of PTSD risk factors, including symptom patterns and severity, explain a large portion of sex differences in PTSD etiology.^14^

DSM-5 criteria enable over 636,000 possible diagnostic combinations of PTSD symptoms, and prior work demonstrates substantial diagnostic overlap between patients.^15,16^ We hypothesized that PTSD symptoms overlap in a sex-specific manner and that this pattern of overlap informs sex-specific disorder etiology. We used genomic structural equations modelling (gSEM) to model multivariate genetic architecture of PTSD symptoms separately in males and females. Using these factor structures, we investigated the wider causal associations between the underlying factors and other heritable traits in the UK Biobank. We report sex-specific factor structures whose genetic architecture differentially overlaps with features of the human phenome that inform sex-based differences in PTSD etiology.

## Methods

### Data Description and Factor Modelling

The UK Biobank (UKB) is a population-based cohort of >500,000 participants. This study used UKB GWAS summary data for posttraumatic stress (PTS) phenotypes from unrelated European ancestry participants adjusted for the first 20 genetic principal components, age, and age^2^.^2^ Sex was not included as a covariate due to the sex-stratified design of this study. Notably, the UKB measured chromosomal and self-reported sex and did not include data on self-reported gender (though participants may have self-reported their gender when asked for their sex). We acknowledge that differences in PTSD diagnoses, personal experience with disorder, and trauma exposure are more relevant for gender as compared to sex. In this study, we use genetic sex as a proxy for gender, which have high, but imperfect, correlation.^17^

gSEM models the multivariate genetic architecture of sets of traits considering their genetic covariance structure.^18^ Using gSEM, a multivariable GWAS was performed with 6 PTS traits based on their unique factor structure in males and females. The 6-factor indicators were questions from the UKB Mental Health Questionnaire (MHQ) and have been previously described by Davis et al. (Table S1).^19^ All initial participants in the UKB were later recontacted and requested to complete the MHQ, for which all participants who opted in (*N* = 157,366) had the option to answer mental health questions. The set of 6 PTSD indicators is based on the abbreviated civilian PTSD checklist (PCL-C), which instructs respondents to answer questions in the context of stressful life experiences.^20^ While the 6-item PCL-C is not equivalent to DSM-5 diagnostic criteria, it is often employed in civilian clinical settings as a screening tool and has been well validated for this purpose.^20^ In the UK Biobank, it is essential to note that the 6-item PCL-C captures general dimensions of PTSD symptoms but is not delivered in the context of a specific traumatic experience. Five questions asked about how often participants were bothered by: (i) *avoiding activities or situations because they remind them of stressors* (“Avoid,” *N* = 117,868, 56% female), (ii) *feeling distant or cut off from others* (“Distant”; *N* = 52,822; 63% female), (iii) *feeling irritable or having angry outbursts* (“Irritable,” *N* = 52,816, 63% female), (iv) *repeated, disturbing memories, thoughts, or images of a stressful experience* (“Thoughts,” *N* = 117,900, 56% female), and (v) *feeling very upset when something reminded them of a stressor* (“Upset,” *N* = 117,893, 56% female). Participants responded to these questions with a rating from 0=“not at all” to 4=“extremely.” The sixth question asked about how bothered participants were by *trouble concentrating on things, such as reading or watching television*, over the last 2 weeks (“Concentrate,” *N* = 117,899, 56% female). Participants responded to this question with a rating from 1=“not at all” to 4=“nearly every day.” For all MHQ survey questions, participants could opt not to answer; these responses are not included in our data.

Multivariable linkage disequilibrium score regression (LDSC) was used to obtain genetic covariance and sampling matrices using a European ancestry LD reference panel from the 1000 Genomes Project. Factor modelling used diagonally weighted least squares estimation and promax rotation. Exploratory factor analysis (EFA) was used to evaluate factor structures for the 6 traits in males and females. We attempted to explore model fit for a 3-factor solution; however, in males and females, the consideration of a third factor extracted a single indicator loading onto that factor. The addition of more factors therefore fails to meaningfully cluster the indicator traits, which may lead to model overfitting. From EFA, confirmatory factor analysis (CFA) was performed using all indicator traits with factor loadings > 0.3.^21,22^ Four model fit statistics were compared to assess the suitability of the models: chi-squared (χ^2^), comparative fit index (CFI), Akaike information criterion (AIC), and standardized root mean square residual (SRMR). The χ^2^ statistic indexes whether modelled genetic covariance differs from an empirical matrix. CFI tests whether the proposed model fits better than a model that assumes all indicators are heritable but uncorrelated. AIC measures relative model fit and may be used to compare multiple models. SRMR measures approximate model fit calculated as the standardized root mean square difference between implied and observed correlations among covariance matrices. Each fit statistic has strengths and weaknesses—we deemed the superior model to have low AIC, low SRMR, and high CFI.

### Comparing Male and Female Factor Structures

Models of PTS in males and females were compared using local standardized root-mean-square difference (localSRMD).^23^ Briefly, localSRMD indexes the extent to which each parameter in the model (i.e., factor loadings) differs across groups, here defined as male and female. To compare across sexes, a covariance matrix was constructed using all PTS indicators from both sexes (twelve total indicators) and subsequently used to estimate unconstrained (all indicators estimated freely) and constrained models (indicators across models forced to be equal). LocalSRMD values are interpretable similar to Cohen's *d*, so localSRMD >0.1 and <0.3 indicated a small difference between models, localSRMD >0.3 and <0.5 indicated a medium difference between models, and local SRMD >0.5 indicated a large difference between models. LocalSRMD < 0.1 indicated a trivial difference. A χ^2^ nested model comparison was performed to test the exact equivalence of parameters by sex. A nontrivial localSRMD and significant χ^2^ (*P* < 0.05) must both be true to render the models appreciably different across sexes.

### Latent Causal Variable Modelling

The latent causal variable (LCV) method was used to identify putative causal relationships between PTS-f_1_ and PTS-f_2_ and each genetically correlated and heritable phenotype from the UKB. Briefly, LCV distinguishes genetic correlation from causation by modelling a latent mediator with a causal effect on each trait.^24^ Furthermore, LCV assumes both traits are genetically correlated, though unaffected by population stratification and linkage disequilibrium. We used a set of pruned SNPs and European-only GWAS summary statistics which adjusted for residual population stratification using PCs for ancestry. For these reasons, we believe our LCV tests satisfied these assumptions.^24^ All genetic causality proportions (gĉps) between 2 traits were estimated with the PTS factor as trait 1 and the UKB trait as trait 2. With this coding in mind, the posterior mean gĉps reported here are interpreted as follows: (i) |gĉp| between 0.7 and 1 indicates a fully causal relationship, (ii) gĉp > 0 indicates that PTS causes trait 2, (iii) gĉp < 0 indicates that PTS is caused by trait 2, and (iv) the causal effect (i.e., “increase” or “decrease” in risk) for significant gĉps were deduced from the genetic correlation estimate.^25,26^ Multiple testing correction was performed using a false discovery rate of 5%. Assessing all traits at an LCV 2-tailed *P* < 0.05, hypergeometric testing was performed to determine enrichment of putatively causal trait domains based on 6 relevant assessments of the data: (i) PTS-f_1_ and PTS-f_2_ cause trait 2; (ii) trait 2 causes PTS-f_1_ and PTS-f_2_; (iii) all traits with significant putative causal effects with PTS-f_1_ and PTS-f_2_ but insignificant gĉp differences measured by 2-sided *Z*-tests, herein listed as “concordant”; (iv) traits with significant gĉps relative to 1 PTS factor and not the other, and significant gĉp differences measured by 2-sided *Z*-tests, herein listed as “discordant”; (v) PTS-f_1_ specific; (vi) PTS-f_2_ specific.

## Results

### Structure of PTS Factors in Males and Females

Common factor and 2-factor models were explored using PTS symptoms in males and females. EFAs suggested 2-factor models fit best in both males (cumulative variance explained for 1-factor model = 0.762, 2-factor model = 0.914) and females (cumulative variance explained for 1-factor model = 0.796, 2-factor model = 0.887; Table S2). While a 3-factor model of PTS symptoms is generally supported in the literature,^16^ a 6-indicator model of PTS symptoms becomes saturated in EFAs that estimate any more than 2 common factors. In other words, in models with 3 or more factors, the variance and covariance of each indicator are perfectly (or near perfectly) reproduced.

**Figure 1.**
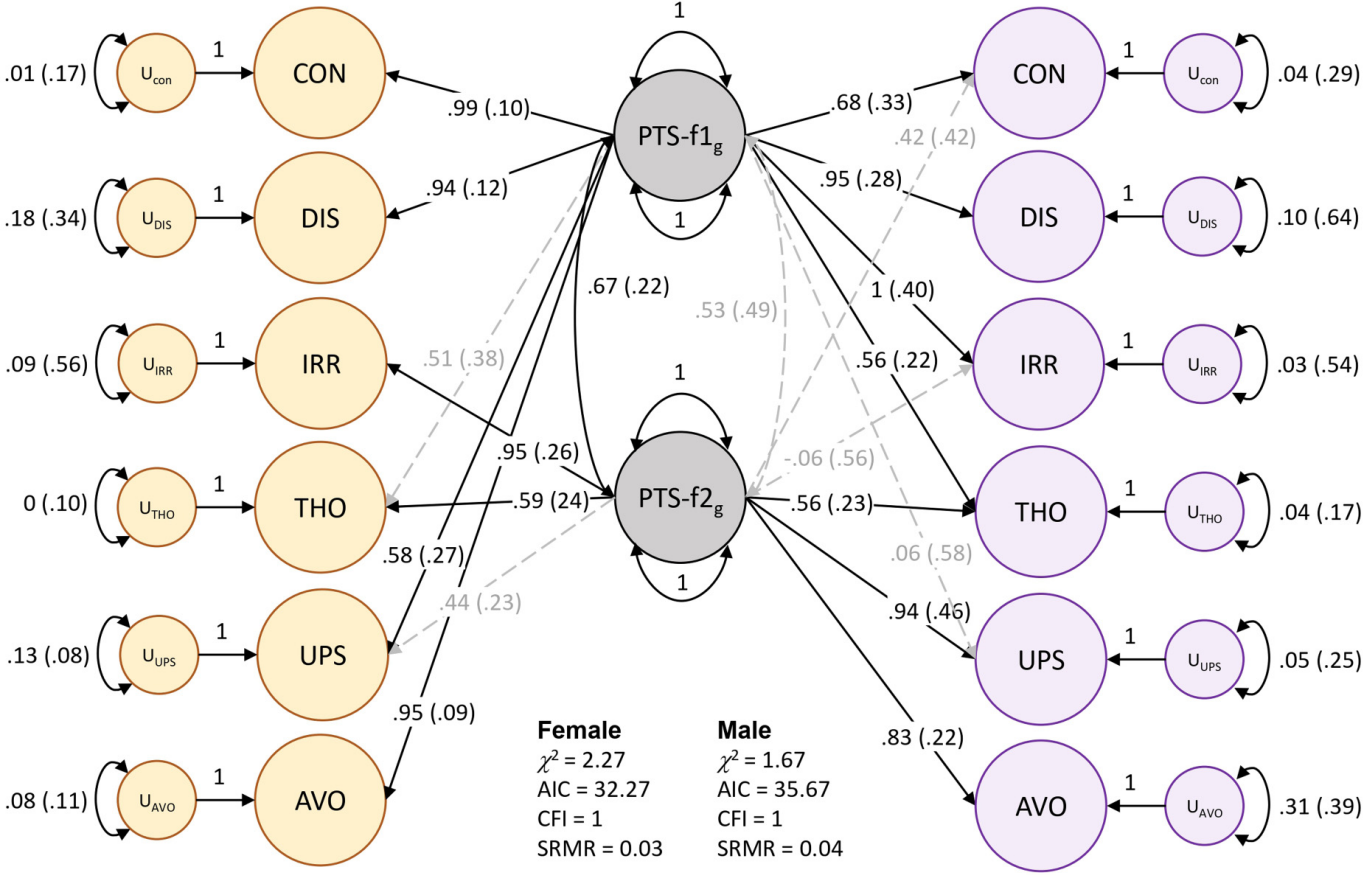
Two-factor model of posttraumatic stress in females (yellow) and males (purple). Standardized loadings are shown for each indicator: CON = trouble concentrating, DIS = feeling distant from others, IRR = irritability, THO = recent disturbing thoughts, UPS = recent upset feelings, AVO = avoidance. The standard error for each loading value is indicated in parentheses. Significant factor loadings (*P* < 0.05) are shown in solid black lines while dashed grey lines show loadings suggested by EFA but did not reach statistical significance (*P* > 0.05) in CFA.

Models of male and female PTS symptoms differed substantially (localSRMD = 3.12) and significantly (χ^2^(5) = 28.03, *P* = 3.6 × 10^−5^). The unstandardized loading for the *thoughts* indicator differed most by sex. Freeing this parameter reduced localSRMD to 2.30, which still indicated a large difference between sexes, but the difference was no longer significant by χ^2^ testing (χ^2^(4) = 9.44, *P* = 0.051). With the relaxed model (i.e., freeing the *thoughts* indicator) we established partial invariance of the factors but were underpowered to detect sex differences in loadings for specific indicators of PTS-f_1_ and f_2_. However, there were noteworthy qualitative patterns that differed by sex. Male PTS-f_1_ largely reflected *irritability* (loading = 1, se = 0.417, *P* = 0.015) and *feeling distant* (loading = 0.95, se = 0.233, *P* = 0.001) while PTS-f_2_ represented *upset feelings* (loading = 0.938, se = 0.462, *P* = 0.042). Female PTS-f1 reflected *trouble concentrating* (loading = 0.995, se = 0.096, *P* = 3.72 × 10^−25^), *avoidance* (loading = 0.957, se = 0.091, *P* = 4.17 × 10^−26^), and *feeling distant* (loading = 0.904, se = 0.120, *P* = 4.68 × 10^−14^) while female PTS-f_2_ represented *irritability* (loading = 0.954, se = 0.258, *P* = 2.16 × 10^−4^). CFAs were constructed for all 4 models (Table S3) and support the separate 2-factor constructions for both males and females (Figure 1, Figure S1, and Table S4). These models were used for the GWAS of each factor.

### Genetic Correlation

After multiple testing correction (FDR < 0.05), 199 of 523 traits were genetically correlated with male PTS-f_1_ and PTS-f_2_, respectively (Table S11). These were enriched for psychiatric traits (2.03-fold, *P* = 1.29 × 10^−14^; Table S12). There were 10 traits associated with male PTS-f_1_ (FDR < 0.05) but not with male PTS-f_2_ (*P* > 0.05). The strongest of these was with *number of full sisters* (UKB Field ID 1883; *r_g_* with male PTS-f_1 _= 0.439, *P* = 0.003 and with male PTS-f_2 _= 0.238, *P* = 0.133). There were 28 traits associated with male PTS-f_2_ (FDR < 0.05) but not with male PTS-f_1_ (*P* > 0.05). These were enriched for cognitive (4.66-fold, *P* = 4.34 × 10^−4^), musculoskeletal (2.66-fold, *P* = 0.022), and nutritional (2.85-fold, *P* = 0.016) trait domains. The strongest of these was with *fluid intelligence* (*positional arithmetic*, UKB Field ID 4968; *r_g_* with male PTS-f_1_=−0.175, *P* = 0.160 and with male PTS-f_2_=−0.477, *P* = 0.002).

After multiple testing correction (FDR < 0.05), there were 274 traits genetically correlated with female PTS-f_1_ and PTS-f_2_ (Table S11). These were enriched for respiratory (1.64-fold, *P* = 9.29 × 10^−12^), gastrointestinal (1.23-fold, *P* = 0.001), and urogenital (1.49-fold, *P* = 0.047) trait domains (Table S12). This set of traits was also depleted for psychiatric traits (7.34-fold, *P* = 2.31 × 10^−7^). There were 29 traits associated with female PTS-f_1_ (FDR < 0.05) but not associated with female PTS-f_2_ (*P* > 0.05). These traits were enriched for body structures (3.64-fold, *P* = 9.67 × 10^−5^)—the strongest result from this trait domain was *whole body water mass* (UKB Field ID 23102; *r_g_* with female PTS-f_1 _= 0.128, *P* = 5.13 × 10^−4^ and with female PTS-f_2 _= 0.074, *P* = 0.056). There were 18 traits associated with female PTS-f_2_ (FDR < 0.05) but not with female PTS-f_1_ (*P* > 0.05). These were enriched for cardiovascular traits (3.64-fold, *P* = 0.036) and ophthalmological traits (8.50-fold, *P* = 0.018)—the strongest genetically correlated trait from these domains was *coronary atherosclerosis* (UKB Field ID I9_CORATHER; *r_g_* with female PTS-f_1 _= 0.146, *P* = 0.126 and with female PTS-f_2 _= 0.219, *P* = 0.023).

### Putative Causal Relationships

**Figure 2.**
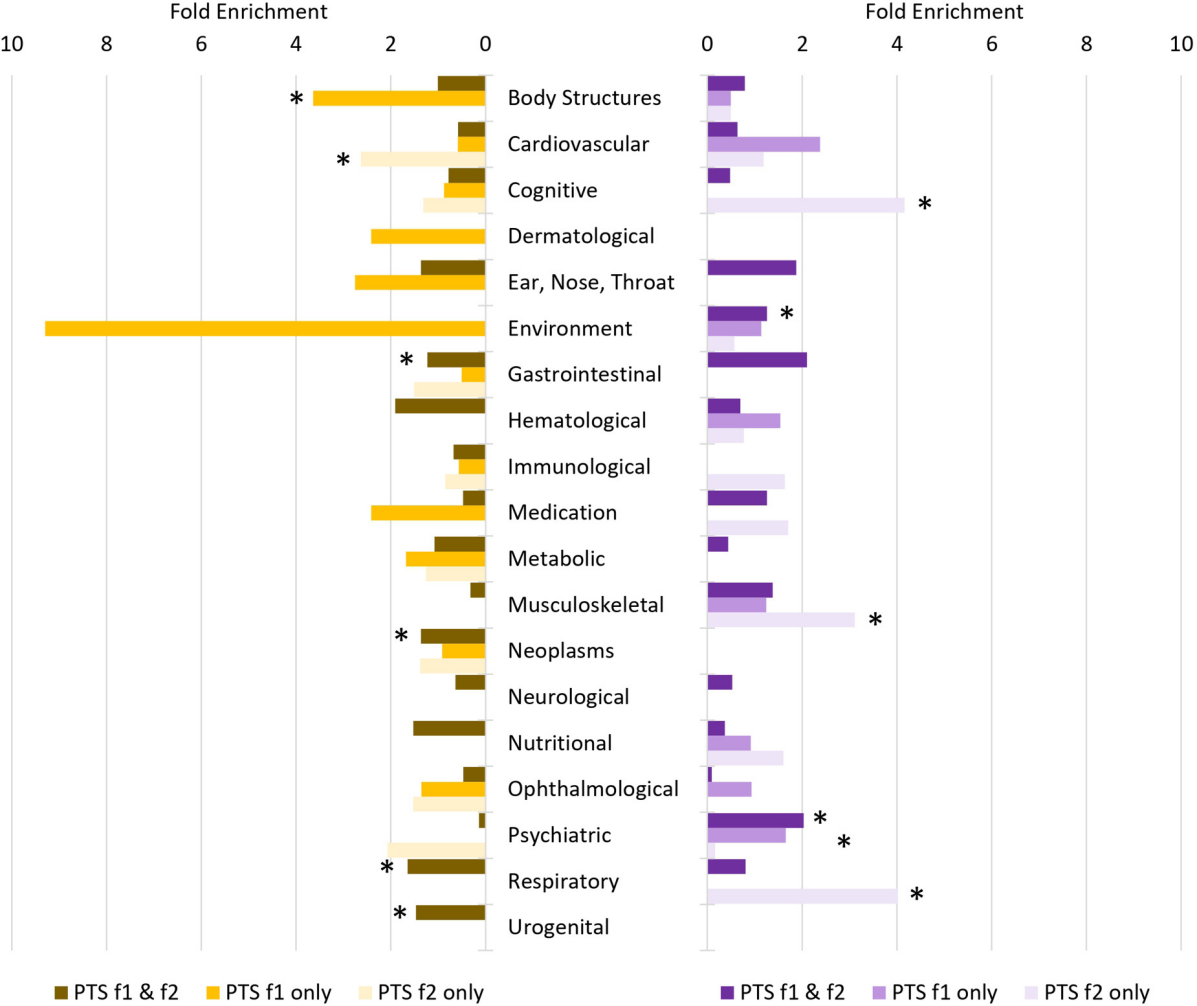
Patterns of trait domain enrichment among genetic correlates of female (left) and male (right) posttraumatic stress (PTS) factors. Fold enrichments were calculated using hypergeometric tests (Supplementary Table S12) with asterisks indicating significant enrichment (*P* < 0.05).

**Figure 3.**
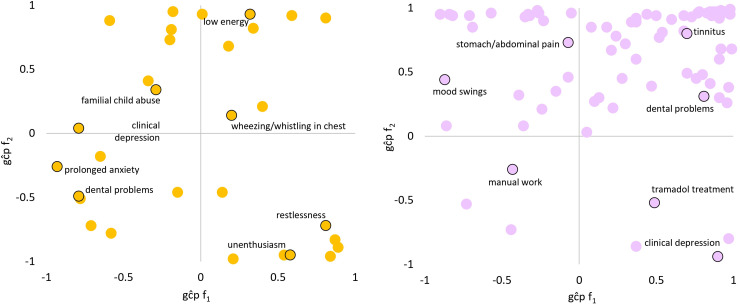
Patterns of significant trait domain genetic causality proportions (gĉps) from latent causal variable analysis of female (left) and male (right). Top-right: caused by f_1_, caused by f_2_. Top-left: causes f_1_, caused by f_2_. Bottom-right: caused by f_1_, causes f_2_. Bottom-left: causes f_1_, causes f_2_. Select traits are highlighted in each quadrant. All gĉp results are shown in Supplementary Tables S13 and S14.

LCV was used to determine if genetic correlations suggested a putatively causal effect mediated by a latent variable (Figure 2; see Figure 3 for specific highlighted traits). After multiple testing correction, there were 99 male PTS-f_1_, 166 male PTS-f_2_, 34 female PTS-f_1_, and 80 female PTS-f_2_ significant putatively causal relationships. Psychiatric traits were enriched for causal estimates with all PTS factors (Tables S13-S15) except the male PTS-f_2_-specific results were depleted for psychiatric traits (3.42-fold depletion, *P* = 8.83 × 10^−7^). Male PTS-f_1_-specific traits were enriched for traits related to medication intake (3.73-fold, *P* = 0.032) while male PTS-f_2_-specific traits were enriched for body structure (2.84-fold, *P* = 5.51 × 10^−7^) and cognitive (3.22-fold, *P* = 0.009) causal relationships. In females, PTS-f_1_-specific traits were enriched in the metabolic domain (28.08-fold enrichment, *P* = 0.035).

## Discussion

Through multivariate modelling of PTSD symptom GWAS and assessment of genetic correlates and putative causal phenotypes, our study identified patterns of phenotypic differences which could inform sex differences in PTSD etiology. Consistent with prior literature, female PTS factors had slightly higher SNP-based *h^2^* estimates than male PTS factors. Looking at factor structure, male PTS factors were uncorrelated while female PTS factors were correlated. These findings may in part be attributed to women reporting more severe PTS symptoms relative to men. Our novel approach to compare male and female factor structures in the UKB indicated that differences in factor loadings of PTSD symptoms do exist across males and females. Further, the underlying genetic architecture differs substantially, as each latent factor has been demonstrated to map differently by sex. Though model comparisons support these differences, we were underpowered to identify which factors differ.^16^ However, the pattern of indicators that load onto each factor show genetic differences between sexes that are reinforced among phenotype-level factor modelling. Overstreet et al. demonstrate among military veterans (92.2% male) that irritability and difficulty concentrating cluster together in a dysphoric arousal factor.^16^ Despite using a civilian cohort to measure PTSD symptoms, our genetic investigation supports this clustering. However, we showed using genetic data that irritability and difficulty concentrating significantly and strongly load onto distinct factors.

Across all 4 GWAS (2 each in males and females), we identified 2 genome-wide significant loci associated with female f_1_: *SCAND3* and *WDPCP* (Supplementary Results). SCAND3 was previously identified as potentially diagnostic for hepatocellular carcinoma, a cancer of the liver.^27^ As cancer may serve as an index trauma, SCAND3 may serve as an indicator of PTS severity, though this relationship requires dedicated testing.^28–30^ We identified *WDPCP* (rs72813410-C) as a risk-decreasing variant for female PTS-f_1_. This gene was previously identified as a risk-increasing genome-wide significant finding in a cross-disorder study of the impulsivity-compulsivity spectrum.^31^ WDPCP is also associated with DNA methylation in past child soldiers^32^ and tinnitus prevalence.^33^ These findings, along with the lack of enrichment for psychiatric genetic correlates and enrichment of putatively causal relationships with body structures, support the identity of female PTS-f_1_ as a general factor of non-psychiatric disease/disorder risk. Despite the lack of individual significant loci identified in the GWAS of female PTS-f_2_, male PTS-f_1_, and male PTS-f_2_, we detail their genetically correlated and putatively causal profiles below.

Male PTS-f_1_ and PTS-f_2_ were genetically correlated with psychiatric traits. Male PTS-f_1_ was not enriched for any genetic correlates but did have specific enrichment of putative causal effects with medication use traits localized to pain relief medications: tramadol (a synthetic opioid), omeprazole (a proton pump inhibitor), and aspirin. Low-dose aspirin is often used for the prevention of myocardial infarction, as well as an over-the-counter analgesic commonly taken by PTSD patients.^34–36^ Patients with PTSD report higher use of opioid and non-opioid analgesics relative to non-PTSD patients.^37^ The relationship between opioid use and PTSD is complex and multidirectional, as opioids may be used to address pain caused by PTSD, while also potentially being related to traumatic events. Our putative causal estimates between pain medication and PTS using the LCV approach were bidirectional and consistent with literature reports of chronic pain and internalizing disorders such as depression.^38^ These findings reinforce several scenarios of their possible causal relationship including: (i) an index trauma causes chronic pain and leads to elevated PTS, (ii) chronic pain (e.g., lower back pain, migraines) itself is a traumatic experience contributing to PTS, and (iii) intrusive and hyperarousal symptoms cause pain complaints and/or pain catastrophizing, respectively.^39^ These relationships are largely considered evidence of shared vulnerability and are reviewed in greater detail by Asmundson et al.^40^ Male PTS-f_2_ was enriched for musculoskeletal traits at the level of genetic correlation and putative causality. Unlike male PTS-f_1_, these results highlight the effects of stress on dental health and mid-to-low back pain.^41,42^ There appear to be pain area-specific and factor-specific effects, but these require further investigation to verify.

LCV analyses uncovered several sex-based differences in PTSD biology, etiology, and symptomatology. While it was expected for psychiatric enrichment to be present in both sexes, the traits within this domain differ. In males, for example, the most significant concordant traits were tiredness/lethargy, trouble relaxing, and low energy, corresponding to depression, anxiety, and sleep-related symptoms. In females, the most significant concordant traits were suffering from nerves, familial abuse as a child, and decreased ability to confide. These findings in females correspond not only to anxiety-related traits, but also treatment, coping mechanisms, and trauma exposure. The genetic causal effects related to familial abuse provide support for studies showing that greater genetic liability for PTSD in women may contribute to earlier onset and/or more severe symptoms, as well as women more generally experiencing trauma early in life.^43^ Furthermore, we found that many of the significantly associated traits in women were anxiety-related (e.g., suffering from nerves, extended periods of anxiety, and tenseness/restlessness were associated with both PTS-f_1_ and PTS-f_2_), supporting findings that women are more prone to anxious symptoms of PTSD.^44,45^ These findings may also simply reflect the increased prevalence of anxiety disorders in women, as well as the strong comorbidity between PTSD and anxiety disorders.^46,47^ Further study of female- and male-specific disorders and symptom factor structures may aid in untangling these observations.

Our study has 3 primary limitations to consider. First, the UKB primarily represents European ancestry individuals of a generally higher socioeconomic bracket. This means that PTS symptoms are likely (i) relatively mild due to the lower prevalence of regular exposure to traumatic events and (ii) reflective of civilian trauma patterns due to the use of the civilian-oriented PCL-6. While genetic evidence suggests no difference in PTSD among civilian and military personnel,^11^ there is evidence that military cohorts are more resilient to trauma,^48^ thereby altering PTSD etiology.^49^ Furthermore, we have previously shown that the UKB combat/war-exposure question influences PTS both directly and interactively with other potentially traumatic events.^50^ For these reasons, our results may only apply to a European civilian population and/or to higher socioeconomic status groups. Second, the UKB does not contain information on the quantity or severity of potentially traumatic events. This information may demonstrate that the PTS symptoms reported by UKB participants are enriched for certain types of experiences; however, sex-stratified GWAS summary data on quantity/frequency of exposure to potentially traumatic experiences are not available to our knowledge. PTS patterns following specific traumas or trauma domains therefore warrant dedicated investigation. Finally, gSEM indicator GWAS used to estimate PTS factor structure fits a linear model to ordinally distributed data. With sample sizes as large as the UKB, this mismatch of model suitability may not be able to control the type I error rate for rarer variants.^51^ Therefore, the genetic signal of the PTS indicators may not fully reflect their true polygenic nature.

In summary, we demonstrate different PTS genetic factor structures between sexes, highlighting the importance of explicitly considering sex in genetic studies of PTSD. By modelling genetic correlation and putative causal relationships of each factor in males and females, we present preliminary data to inform how different dimensions of PTS may influence other traits, behaviours, and disorders. These data inform the etiological differences between males and females supported by existing literature and introduce new hypotheses to help understand the biology of PTS and PTSD. Our findings may further indicate that gene-by-environment interactions related to PTS symptom presentation may differ by sex and the specific constellation of symptoms underlying those differences may inform co-occurring conditions.

## Supplemental Material

sj-docx-1-cpa-10.1177_07067437241301016 - Supplemental material for Sex-stratified Genomic Structural Equation Models of Posttraumatic Stress Inform PTSD Etiology: L'utilisation de la modélisation génomique par équations structurelles stratifiée par sexe du stress post-traumatique pour expliquer l'étiologie du TSPT Supplemental material, sj-docx-1-cpa-10.1177_07067437241301016 for Sex-stratified Genomic Structural Equation Models of Posttraumatic Stress Inform PTSD Etiology: L'utilisation de la modélisation génomique par équations structurelles stratifiée par sexe du stress post-traumatique pour expliquer l'étiologie du TSPT by Ashley Moo-Choy, Murray B. Stein, Joel Gelernter and Frank R. Wendt in The Canadian Journal of Psychiatry

sj-xlsx-2-cpa-10.1177_07067437241301016 - Supplemental material for Sex-stratified Genomic Structural Equation Models of Posttraumatic Stress Inform PTSD Etiology: L'utilisation de la modélisation génomique par équations structurelles stratifiée par sexe du stress post-traumatique pour expliquer l'étiologie du TSPT Supplemental material, sj-xlsx-2-cpa-10.1177_07067437241301016 for Sex-stratified Genomic Structural Equation Models of Posttraumatic Stress Inform PTSD Etiology: L'utilisation de la modélisation génomique par équations structurelles stratifiée par sexe du stress post-traumatique pour expliquer l'étiologie du TSPT by Ashley Moo-Choy, Murray B. Stein, Joel Gelernter and Frank R. Wendt in The Canadian Journal of Psychiatry
