# Supplementary material for: Sex-stratified Genomic Structural Equation Models of Posttraumatic Stress Inform PTSD Etiology: L'utilisation de la modélisation génomique par équations structurelles stratifiée par sexe du stress post-traumatique pour expliquer l'étiologie du TSPT
Source: Can J Psychiatry. 2024 Dec 9;70(2):117–26. doi: 10.1177/07067437241301016 (PMC11629358; doi:10.1177/07067437241301016)
Supplement: sj-docx-1-cpa-10.1177_07067437241301016 - Supplemental material for Sex-stratified Genomic Structural Equation Models of Posttraumatic Stress Inform PTSD Etiology: L'utilisation de la modélisation génomique par équations structurelles stratifiée par sexe du stress post-traumatique pour expliquer l'é [file sj-docx-1-cpa-10.1177_07067437241301016.docx]

# **SUPPLEMENTARY METHODS**

**Annotating PTS Factor GWAS**

GWAS for PTS factors 1 (PTS-f_1_) and 2 (PTS-f_2_) were annotated using Multi-marker Analysis of GenoMic Annotation (MAGMA v1.08) implemented in FUMA v1.5.3 using 2-kb positional mapping around each lead SNP. Linkage disequilibrium independent loci were defined by lead SNPs with p<5x10^-8^ and surrounding SNPs with *r^2^*>0.6 with the lead SNP. Enrichment of tissue transcriptomic profiles was tested using to GTEx v8 for investigation of 54 general tissue types and BrainSpan for investigation of developmental stage and age of brain tissues. Multiple testing correction was applied using FDR (5%) considering all tissue-types or cell-types tested in both sexes. Genome-wide significant SNPs had association p<5x10^-8^ while genome-wide significant genes had association p<2.58x10^-6^ based on a Bonferroni correction for the 19,365 protein-coding genes mapped by FUMA-MAGMA. Significant SNPs and genes for each PTS factor were subjected to phenome-wide association testing in the GWAS Atlas PheWAS browser. Because pleiotropy is widespread and the GWAS Atlas contains many highly correlated traits, we chose to investigate enrichment of associated trait domains rather than lists of correlated single traits. Enrichment of associated phenotypes was determined using hypergeometric tests of 4,756 traits separated into 25 trait domains.

For each genome-wide significant SNP and annotated gene, we performed a phenome-wide association study in the GWAS Atlas. Because pleiotropy is widespread and the GWAS Atlas contains many highly correlated traits, we chose to investigate enrichment of associated trait domains rather than lists of correlated single traits. Enrichment of associated phenotypes was determined using hypergeometric tests of 4,756 traits separated into 25 trait domains: activities (N=137), aging (N=5), body structures (N=18), cardiovascular (N=162), cellular (N=1,143), cognitive (N=78), connective tissue (N=14), dermatological (N=31), ear, nose, and throat (N=6), endocrine (N=67), environmental (N=92), gastrointestinal (N=42), hematological (N=38), immunological (N=365), infection (N=3), metabolic (N=1,259), mortality (N=46), muscular (N=5), neoplasms (N=49), neurological (N=437), nutritional (N=46), ophthalmological (N=28), psychiatric (N=321), reproduction (N=76), respiratory (N=74), skeletal (N=194), and social interactions (N=20).

**Linkage Disequilibrium Score Regression (LDSC)**

LDSC was used to estimate the *h^2^*-SNP of PTS-factors in each sex and in publicly available sex-stratified GWAS summary association data from the UKB using the 1000 Genomes Project European ancestry reference panel. The major histocompatibility complex region was excluded from these analyses due to its complex linkage disequilibrium structure. In UKB, 523 had an *h^2^*-SNP Z-score>4 in both sexes deeming them appropriate for inclusion in cross-trait genetic correlation (*r_g_*) with PTS-factors in this study. Two-sided Z-tests were used to compare *r_g_* estimates between the two factors within each sex. Traits correlated with one factor (FDR<0.05) but not with the other factor (P>0.05) *and* with a significant difference between r_g_ were considered “specific” to that factor.

# **SUPPLEMENTARY RESULTS**

**Genetic architecture of male and female PTS factors**

There were two loci associated with female PTS-f_1_: (i) rs146918648 (β=-0.072, p=6.73x10^-9^) positionally mapped to an intronic region of *SCAND3*, previously detected in studies of well-being, depressive symptoms, and positive affect (Tables S5 and S6) and (ii) rs72813410-C (β=-0.08, P=1.94x10^-8^) which positionally mapped to a intronic region of *WDPCP*, previously detected in studies of smoking initiation and age of first sexual intercourse (Tables S7 and S8). Gene-based association testing of female PTS-f_1_ revealed an association with *FAM120A* (Z=4.56, P=2.52x10^-6^), which has been detected previously in studies of neuroticism, loneliness, and comparative body size at age 10 (Table S9). The GWAS for female PTS-f_1_ was enriched for transcriptomic effects from spinal cord cervical c-1 (β=0.015, p=0.035), hippocampus (β=0.013, p=0.039), hypothalamus (β=0.013, p=0.040), and substantia nigra (β=0.013, p=0.049; Table S10), while the GWAS of female PTS-f_2_ was enriched for vaginal transcriptomic effects (β=0.018, p=0.046). There were no genome-wide significant loci associated with either male PTS factor (Table S10).

**Putative Causal Relationships**

We identified 99 male PTS-f_1_, 166 male PTS-f_2_, 34 female PTS-f_1_, and 80 female PTS-f_2_ significant putatively causal relationships. Of the medication-related traits specifically enriched in male PTS-f_1_, we observed conflicting putative causal estimates with respect to omeprazole use. General omeprazole use was putatively causal for male PTS-f_1_ (gĉp=-0.05, p=0.005); however, omeprazole use ascertained in the context of pain relief, constipation, and/or heartburn was caused by PTS-f_1_ at much larger magnitude (gĉp=0.39, p=0.020). The body structure traits with putative causal effects relative to male PTS-f_2_ were mainly for measures of body fat percentages (including whole body, arms, legs, and waist), as well as body mass index (BMI; gĉp=0.8; p=3.41x10^-8^) increasing as a result of PTS-f_2_. Additionally, in the cognitive domain, PTS-f_2_ had causal effects with decreased performance on fluid intelligence (gĉp=0.56; p=1.53x10^-6^) and reaction time (gĉp=0.89; p=6.24x10^-10^) tests. The most significant male PTS-f_2_-specific findings indicated that PTS-f_2_ causes repeated disturbing thoughts of stressful experiences (gĉp=0.9; p=6.24x10^-10^) and increased risk-taking behaviour (gĉp=0.44; p=2.48x10^-8^). In females, enrichment was detected for metabolic traits in PTS-f_1_, in which the PTS-f_1_-specific association was high cholesterol having a causal effect on PTS-f_1_ (gĉp=-0.78; p=1.04x10^-7^). Female PTS-f_2_ had many specific putative causal relations with psychiatric traits; the most significant of which were anxiety and depression-related: PTS-f_2_ causing nervous feelings (gĉp=-0.53; p=2.16x10^-32^) and extended periods of unenthusiasm/disinterest (gĉp=-0.95; p=3.13x10^-30^) and tiredness/lethargy causing PTS-f_2_ (gĉp=-0.91; p=5.09x10^-21^).

# **SUPPLEMENTARY FIGURES**


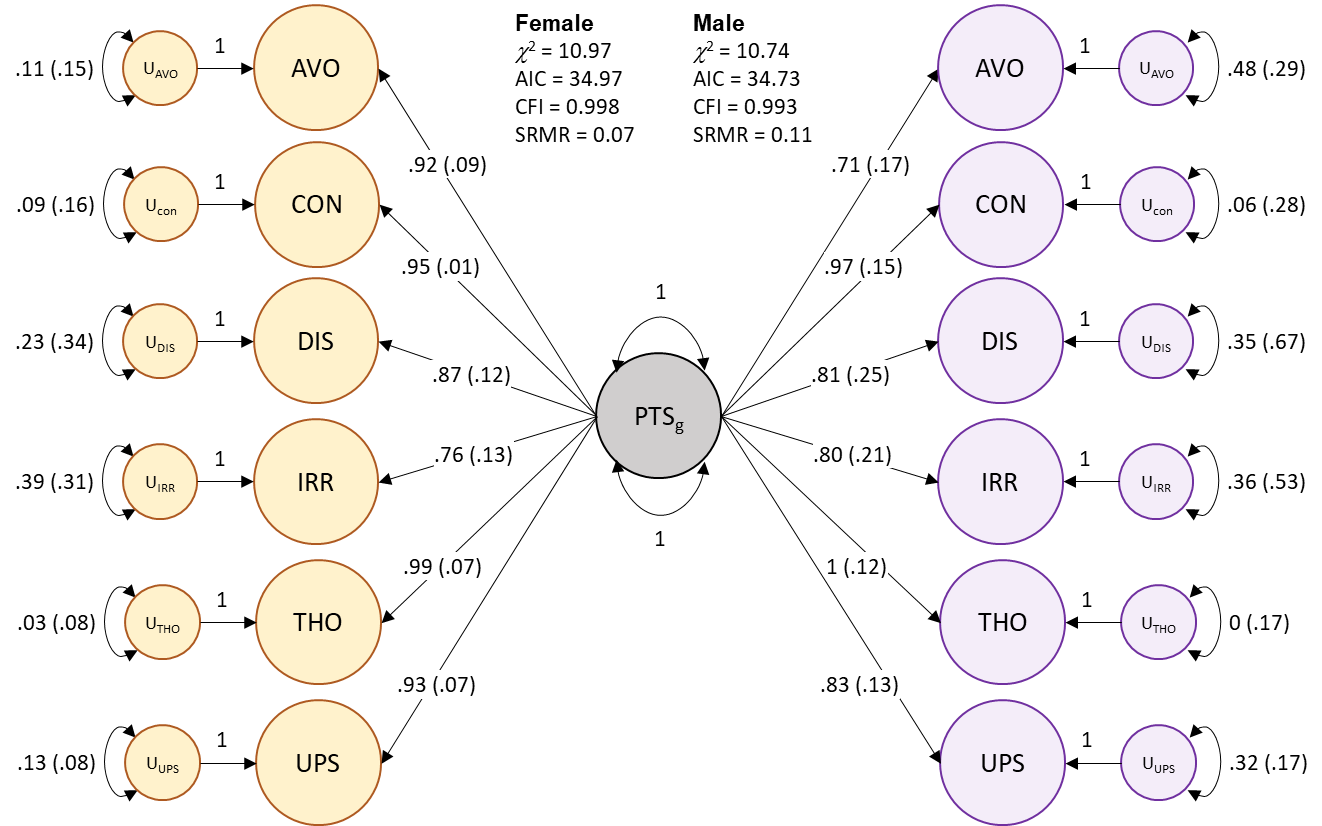


**Figure S1.** Common factor model of posttraumatic stress in females (yellow) and males (purple). Standardized loadings are shown for each indicator: CON = trouble concentrating, DIS = feeling distant from others, IRR = irritability, THO = recent distributing thoughts, UPS = recent upset feelings, AVO = avoidance. All factor loadings were significant (p<0.05).

# **SUPPLEMENTARY TABLES**

**Table S1.** Trait description and observed-scale heritability estimates for each posttraumatic stress indicator. Heritability comparisons between males and females were calculated using two-sided Z-tests.

**Table S2.** One- and two-factor exploratory factor analysis results for posttraumatic stress indicators. Highlighted traits were included in confirmatory factor analyses.

**Table S3.** Confirmatory factor fit statistics for each factor structure tested. CFI = comparative fit statistics, AIC = Akaike information criterion, SRMR = standardized root mean square residual.

**Table S4.** Loading values for each indicator on the two-factor model stratified by sex.

**Table S5.** GWAS Atlas PheWAS results for female PTS-f_1_ locus rs146918648.

**Table S6.** GWAS Atlas PheWAS results for female PTS-f_1_ locus *SCAND3*.

**Table S7.** GWAS Atlas PheWAS results for female PTS-f_1_ locus rs72813410.

**Table S8.** GWAS Atlas PheWAS results for female PTS-f_1_ locus *WDPCP*.

**Table S9.** GWAS Atlas PheWAS results for female PTS-f_1_ locus *FAM120A*.

**Table S10.** Enrichment of brain transcriptomic profiles in the GWAS for female PTS-f_1_. Highlighted tissues are nominally significant (p<0.05).

**Table S11.** Genetic correlation estimates between each PTS factor and a UK Biobank trait with a heritability Z-score > 4. Comparisons between factors (within each sex) were performed using two-sided Z-tests.

**Table S12.** Results of hypergeometric enrichment tests for genetic correlation results (Table S11) for traits associated with both factors and traits uniquely associated with one trait. Highlighted trait domains are significantly enriched.

**Table S13.** Genetic causality proportion estimates between male PTS factors and a UK Biobank trait. Comparisons between factors (within each sex) were performed using two-sided Z-tests.

**Table S14.** Genetic causality proportion estimates between female PTS factors and a UK Biobank trait. Comparisons between factors (within each sex) were performed using two-sided Z-tests.

**Table S15.** Results of hypergeometric enrichment tests for LCV results for all categories (PTS-f_1_ and PTS-f_2_ cause trait 2, trait 2 causes PTS-f_1_ and PTS-f_2_, concordant, discordant, PTS-f_1_-specific, PTS-f_2_-specific). Highlighted trait domains are significantly enriched.
